# Neutrophil killing of *Staphylococcus aureus* in diabetes, obesity and metabolic syndrome: a prospective cellular surveillance study

**DOI:** 10.1186/s13098-017-0276-3

**Published:** 2017-10-03

**Authors:** Ingrid Lea Scully, Lisa Kristin McNeil, Sudam Pathirana, Christine Lee Singer, Yongdong Liu, Stanley Mullen, Douglas Girgenti, Alejandra Gurtman, Michael W. Pride, Kathrin Ute Jansen, Paul L. Huang, Annaliesa S. Anderson

**Affiliations:** 1 Pfizer Vaccine Research and Development, 401 North Middletown Rd, Pearl River, NY 10965 USA; 2 Cardiovascular Research Center and Cardiology Division, Department of Medicine, Massachusetts General Hospital and Harvard Medical School, Boston, MA USA

**Keywords:** Diabetes, Immune function, Metabolic syndrome, Neutrophils, Obesity, *Staphylococcus aureus*, Vaccine

## Abstract

**Background:**

Obesity, metabolic syndrome (MetS), and diabetes are frequent in surgical populations and can enhance susceptibility to postoperative surgical site infections. Reduced neutrophil function has been linked with diabetes and risk of *Staphylococcus aureus* infection. Therefore, neutrophil function in diabetic and obese subjects (± MetS) was assessed in this prospective serological and cellular surveillance study to determine whether vaccines administered to protect against infections after surgery could be effective in these populations.

**Methods:**

Neutrophil function (chemotaxis, phagocytosis, and opsonophagocytic killing of *S. aureus*) was assessed in subjects classified according to diabetes status, body mass index, and presence/absence of MetS. Neutrophils were characterized within functional subsets by flow cytometry. A serologic assay was used to measure baseline antibody presence to each antigen in SA4Ag: capsular polysaccharide (CP) type 5, CP8, recombinant mutant Clumping factor A (rmClfA), and recombinant Manganese transport protein C (rMntC).

**Results:**

Neutrophil function was similar for comorbid and healthy cohorts, with no significant between-group differences in cell counts, migration, phagocytosis ability, neutrophil subset proportions, and *S. aureus* killing ability when neutrophils were isolated 3–6 months apart (Visit 1 [n = 90] and Visit 2 [n = 70]) and assessed. Median pre-existing antibody titers to CP5, CP8, and rmClfA were comparable for all cohorts (insufficient subjects with rMntC titers for determination).

**Conclusions:**

MetS, diabetes, and obesity do not impact in vitro neutrophil function with regard to *S. aureus* killing, suggesting that if an effective *S. aureus* vaccine is developed it may be effective in individuals with these comorbidities.

## Background

Obesity, metabolic syndrome, and diabetes are frequent comorbid disorders in surgical populations, which may enhance patients’ susceptibility to postoperative surgical site infections. The Gram-positive bacterium *Staphylococcus aureus* is a bacterial pathogen that frequently causes healthcare-associated infections, especially among adults undergoing major surgery. Invasive staphylococcal infections are more prevalent in patients with diabetes and obesity than in those without, and are associated with a poor outcome [1–3].

The underlying mechanisms linking these comorbidities to *S. aureus* infection are not fully defined, but may be linked to impairment in several aspects of the immune response to bacterial infections. These aspects include impaired healing, fibroblast and epidermal cell dysfunction, impaired angiogenesis, damage from reactive oxygen species and advanced glycation end products, and decreased host immune resistance [4]. The primary defense against gram-positive pathogens such as *S. aureus* is engulfment and oxidative killing by neutrophils, a process that is dependent on tissue oxygen tension. Obese patients have decreased tissue oxygen tension and poor blood supply. In those undergoing surgery, this presents a particular problem at the surgical incision site, and increases the risk for surgical site infections [5]. Decreased serum and tissue concentrations of prophylactic antibiotics and increased rates of perioperative hyperglycemia [6] may further increase the risk of postoperative infection.

There are reports of impaired bactericidal functions, including phagocytosis, adhesion to endothelium, and chemotaxis by neutrophils in patients with diabetes [7–9]. Conversely, other reports have failed to show significant differences in immunological function in patients with diabetes versus healthy patients [10]. Impaired peripheral blood mononuclear cell (PBMC) function, decreased lymphocyte proliferation, and altered peripheral cytokine levels have also been reported in patients with obesity [11].

Distinct subsets of circulating neutrophils in peripheral blood, based on maturity, have been described during acute systemic inflammation. These cells may also differ in their functional capacities, such as chemotaxis and adhesion characteristics [12, 13].

In diabetic mouse models, chronic wounds are characterized by the presence of elevated cytokines, increased neovascularization, and infiltration of inflammatory cells such as macrophages and neutrophils [14, 15]. Manifestations of neutrophil dysfunction such as decreased phagocytosis, superoxide production, and killing activity of *S. aureus* have also been observed in diabetic *db/db* mice [16].

The challenges of controlling *S. aureus* infections as well as the associated treatment costs are exacerbated by increasing rates of resistance to available antibiotics. Currently, there is no licensed, prophylactic *S. aureus* vaccine that can prevent postoperative infections in high-risk patients. Such a vaccine could help to reduce the incidence of *S. aureus* disease and the associated morbidity, mortality, and cost.

The results of previous unsuccessful vaccine development programs and preclinical research programs indicate that an effective vaccine against *S.* *aureus* should contain several antigens targeting multiple virulence mechanisms  [17, 18]. A prophylactic *S. aureus* 4-antigen (SA4Ag) vaccine is under evaluation in a Phase IIb trial (NCT02388165) in adults undergoing elective spinal fusion. The SA4Ag vaccine is composed of 2 capsular polysaccharide conjugates (CP5-CRM_197_ and CP8-CRM_197_), recombinant surface protein clumping factor A (rmClfA) and recombinant MntC (rMntC) from the ligand binding portion of lipoprotein manganese transporter C. rMntC facilitates *S. aureus* survival in vivo, and preclinical evaluations supported the addition of rMntC to target this bacterial virulence factor [19].

In a dose-ranging, Phase I, randomized, placebo-controlled, clinical study in healthy adults, the precursor to the SA4Ag vaccine, a non-adjuvanted 3-antigen *S. aureus* vaccine (SA3Ag), which included CP5-CRM_197_, CP8-CRM_197_, and rmClfA, was found to induce robust, functional (bacteria-killing) immune responses, with an acceptable safety and tolerability profile [20]. These immune responses were maintained through 12 months after a single vaccination [20]. Based on the immunogenicity and safety findings of this study, 30 µg CP5-CRM_197_, 30 µg CP8-CRM_197_, and 60 µg rm*C*lfA were selected for inclusion in the SA4Ag formulation. Two Phase I/Phase II studies in healthy adults showed that SA4Ag was well tolerated and induced rapid and robust functional immune responses to all 4 antigens after a single vaccination. Antibody levels remained substantially above pre-vaccination levels through month 12 following vaccination [21, 22]. The dose level of rMntC for inclusion in the final formulation of SA4Ag was 200 µg, based on dose level-dependent immune responses to rMntC and the overall safety profile shown in these studies [21, 22].

Neutrophils provide an essential primary defense against *S. aureus*, and are therefore likely to contribute to vaccine-mediated, protective, immune responses. To provide a better understanding of the likelihood that an *S. aureus* vaccine could be effective in subjects with diabetes, obesity, and metabolic syndrome (MetS), neutrophil functions in these patient populations were evaluated in this prospective serological and cellular surveillance study. The primary objectives of this study were to descriptively compare neutrophil function in six cohorts of adult subjects: (1) adults with well-controlled diabetes mellitus, (2) adults with poorly controlled (hemoglobin A1c (HbA1c) ≥ 10%) diabetes mellitus, (3) adults with morbid obesity (body mass index, BMI ≥ 40 kg/m^2^), (4) obese adults (BMI ≥ 30 kg/m^2^) with MetS, (5) obese adults without MetS, and (6) healthy patients with normal BMI (18.5–24.9 kg/m^2^) and without diabetes mellitus.

Secondary objectives were to descriptively compare immune function in: adults with well-controlled (HbA1c < 7%) and poorly-controlled (HbA1c ≥ 10%) diabetes mellitus; adults without diabetes mellitus and with well-controlled diabetes mellitus (HbA1c < 7%); obese adults (BMI 30 to < 40 kg/m^2^) and morbidly obese adults (BMI ≥ 40 kg/m^2^). Neutrophil function was evaluated with regard to chemotactic migration, bacterial phagocytosis and opsonophagocytosis (bacterial killing). Neutrophil subsets (normal, killer, and suppressor) and plasma antibody titers were also assessed.

## Methods

### Study design and patient selection

This was an exploratory clinical research collaboration between Massachusetts General Hospital and the Pfizer Vaccine Research and Development Unit. All subjects were interviewed and received physical examinations and laboratory testing. Written informed consent was obtained from all participants before study procedures were initiated. The study was approved by the Institutional Review Board of the Massachusetts General Hospital.

Subjects were not vaccinated for this study. Two fasting blood draws (Visit 1 and Visit 2) were taken approximately 3–6 months apart for immune function assays. At Visits 1 and 2, 50 mL blood was drawn for evaluation of immune function, and 10 mL blood was drawn for fasting lipid profile, fasting blood glucose, C-reactive protein (CRP), and HbA1c. Based on the results from these evaluations at Visit 1 blood draw, all subjects (or a subset) from each cohort were selected for Visit 2 blood draw.

In the main analysis, MetS was diagnosed according to the National Cholesterol Education Program Adult Treatment Panel III (NCEP ATPIII) criteria when any 3 or more of the 5 criteria below were met [23]:

1. Waist circumference > 35 in. in women or > 40 in. in men.
2. Triglycerides > 150 mg/dL.
3. HDL-cholesterol < 50 mg/dL in women or < 40 mg/dL in men.
4. Blood pressure > 130/85 mmHg.
5. Fasting glucose > 100 mg/dL.

### Inclusion criteria

**Fig. 1:**
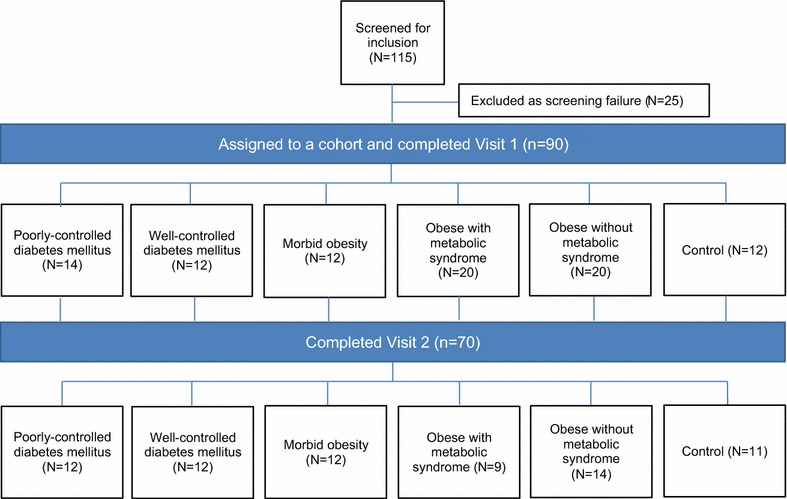
Patient disposition

Subjects were included in the study if they were aged 30–75 years and fulfilled any of the following 6 cohort groups; the target was at least 12 subjects per subgroup, based on the following criteria and described in Fig. 1.

- *Poorly controlled diabetes mellitus cohort*: 12 subjects with diabetes mellitus and HbA1c ≥ 10% (BMI < 30 kg/m^2^). Priority was given to subjects with HbA1c ≥ 10% and BMI < 30 kg/m^2^. Where sufficient subjects presenting with HbA1c ≥ 10% could not be identified, the investigator preferentially selected the candidate subjects with the highest HbA1c levels ≥ 8.5% to fill the cohort. When sufficient subjects with a BMI < 30 kg/m^2^ could not be identified, subjects were prioritized by the lowest BMI values < 40 kg/m^2^ to fill the cohort.
- *Well controlled diabetes mellitus cohort*: 12 subjects with diabetes mellitus and HbA1c < 7% (BMI < 30 kg/m^2^).
- *Morbid obesity cohort*: 12 subjects with morbid obesity (BMI ≥ 40 kg/m^2^), no diabetes mellitus and HbA1c < 6%, or well-controlled diabetes and HbA1c < 7%.
- *Obese with MetS cohort*: 12 subjects with BMI 30 to < 40 kg/m^2^ and a diagnosis of MetS, no diabetes mellitus and HbA1c < 6%, or well-controlled diabetes and HbA1c < 7%.
- *Obese without MetS cohort*: 12 subjects with BMI 30 to < 40 kg/m^2^, with clinical assessments and laboratory data not consistent with MetS, no diabetes mellitus and HbA1c < 6%, or well-controlled diabetes and HbA1c < 7%. When recruiting subjects to this cohort, priority was given to subjects with no diabetes mellitus and HbA1c < 6%.
- *Healthy patient control cohort*: 12 subjects without a diagnosis of diabetes mellitus and HbA1c < 6.0%, without MetS, and with normal BMI (18.5–24.9 kg/m^2^).

### Exclusion criteria

Exclusion conditions included: inability to give blood, self-reported diseases (hepatitis B, hepatitis C, human immunodeficiency virus [HIV]), end-stage renal or liver disease, or malignancy that was treated), immunocompromised status, other severe acute or chronic medical or psychiatric condition or laboratory abnormality that may increase the risk associated with study participation or may interfere with the interpretation of study, participation in other interventional or investigational studies within 30 days before the current study through study completion, receipt of blood products or immunoglobulins within 6 months, and receipt of antibiotic therapy within 72 h of blood draw, pregnancy, or surgery within 30 days of blood draw.

### Immunogenicity measurements

Neutrophils and peripheral blood mononuclear cells (PBMCs) were isolated from collected fasting blood samples. Briefly, after Ficoll-Paque (Fisher) centrifugation of peripheral blood, neutrophils and PBMCs were separated from erythrocytes by 3% dextran-500 (Sigma) density-gradient sedimentation. Isolated cells were resuspended in Hank’s Balanced Salt Solution (Cellgro) for cell counts. The in vitro assays described below were used to measure functional responses to *S. aureus*. Isolated PBMC and neutrophil cell counts were expressed as cell count × 10^7^.

#### Chemotaxis assay

The chemotaxis assay measured in vitro migration of isolated neutrophils towards the test chemoattractants, *N*-formyl-methionyl-leucyl-phenylalanine (fMLP), interleukin (IL)-8, complement component C5a, fetal bovine serum (FBS), and RPMI media. Results were expressed as % migration, calculated as the proportion of signal in the test wells compared to directly lysed input cell control, which was set at 100%. Chemotaxis was measured using a 96-well cell migration assay kit (Cell BioLabs, cat # CBA-104) with a polycarbonate membrane plate to determine the migratory properties of the cells. Any migratory cells were first dissociated from the membrane, then lysed and detected with CyQuant GR Dye provided with the kit, per the manufacturer’s instructions.

#### Phagocytosis assay

The phagocytosis assay (pHrodo assay) measured in vitro neutrophil uptake of opsonized *S. aureus*. Assay results were expressed as % phagocytosis, representing the % of cells that had fluorescent signal. The assay utilizes a pH-sensitive, rhodamine-based pHrodo™ Red dye (Life Technologies, cat # P3660) to detect neutrophil phagocytic functionality. pHrodo fluoresces when exposed to low pH (< 4.0), which is required for bactericidal functions and antigen processing and presentation. Phagocytosis is therefore quantifiable when the phagosome forms and acidifies around ingested pHrodo-labeled *S. aureus*, minimizing high background fluorescence of cell-bound but uningested bacteria [24, 25]. The opsonizing agents were individual (autologous) plasma samples, normal human sera, and human CP8 immune sera. Uptake in the absence of added serum was also tested as a control (no opsonization).

#### Neutrophil phenotype subset assay

The isolated neutrophils were sorted into three different subsets based on CD16 and CD62L expression using flow cytometry: normal (CD16^bright^ CD62L^bright^), suppressor (CD16^bright^ CD62L^dim^), and killer (CD16^dim^ CD62L^bright^) neutrophils, as previously described [13]. CD47 expression was also measured. Results were expressed as % cells for the subsets, based on the % of cells falling into a flow cytometric gate for each subset, and mean fluorescence intensity (MFI) for the CD47 assay.

#### Antibody-mediated opsonophagocytic assay

Functional immune responses were determined in opsonophagocytic activity (OPA) killing assays with isolated neutrophils and clinical *S. aureus* strains expressing CP8. Neutrophils were tested with 3 different human CP8 immune sera (high-titered, medium-titered, and low-titered sera) [26]. Results were expressed as OPA titers, defined as the serum dilution that killed 50% of the input bacteria in the assay in a complement and effector cell dependent manner.

#### Competitive Luminex immunoassay (cLIA)

A 4-plex competitive Luminex^®^ (Luminex Corporation, Austin, TX, USA) immunoassay (cLIA) that measures the ability of serum immunoglobulin to compete with the binding of antigen-specific monoclonal antibodies to antigen-coated microspheres was used to detect the pre-existing CP5, CP8, ClfA, and rMntC antibody titers in the selected patient cohorts [20, 27]. Results were determined against a reference standard and expressed as cLIA titers.

### Alternative AHA/NHLBI diagnostic criteria for metabolic syndrome

The diagnostic criteria outlined by the 2005 American Heart Association/National Heart, Lung, and Blood Institute (AHA/NHLBI) for MetS [28] was used to analyse the subject data in *a* post hoc analysis. The main difference between the AHA/NHLBI and the NCEP ATPIII criteria is that according to the AHA/NHLBI criteria definition, MetS is diagnosed if an individual has normal laboratory/blood pressure values but is receiving drug treatment for the condition while the NCEP ATPIII criteria does not include these patients.

Using the AHA/NHLBI criteria, MetS was diagnosed when any three or more of the following criteria were met [28]:

1. Waist circumference ≥ 35 in. in women or ≥ 40 in. in men.
2. Triglycerides ≥ 150 mg/dL or on drug treatment for elevated triglycerides.
3. HDL-cholesterol < 50 mg/dL in women or < 40 mg/dL in men or on drug treatment for reduced HDL-cholesterol.
4. Blood pressure ≥ 130/85 mmHg or on antihypertensive drug treatment (patient with a history of hypertension).
5. Fasting glucose ≥ 100 mg/dL or on drug treatment for elevated glucose.

### Statistical analysis

For continuous variables, the number (n), mean, median, standard deviation (SD), minimum (min) and maximum (max) for normally distributed endpoints or n, geometric mean, min, max, % relative standard deviation (RSD) and 95% confidence intervals (CI) for log-normally distributed endpoints were summarized using descriptive statistics. For categorical variables, n, percentage, and total (N) were also summarized using descriptive statistics. The descriptive statistics as detailed above were used for all neutrophil analyses.

## Results

### Subject disposition and characteristics

A total of 115 participants were screened, of whom 90 were assigned to a cohort and completed Visit 1, and 70 completed Visit 2 (Fig. 1). The remaining 20 were unable to be scheduled for Visit 2. The demographic characteristics of all 6 cohorts were comparable except for the gender distribution (Table 1). There were similar proportions of males and females in the well-controlled diabetes (50%) and morbidly obese (50%) cohorts; more males in the poorly-controlled diabetes (71%: 29%), obese with MetS (70%: 30%) and obese without MetS (60%: 40%) cohorts; and more females in the healthy patient control cohort (33%: 67%). The mean age across all cohorts was 58.9 years and the majority were white (Table 1).

**Table 1:** Subject demographics

Cohort						
Characteristics	Poorly-controlled diabetes mellitus (N = 14)	Well-controlled diabetes mellitus (N = 12)	Morbid obesity (N = 12)	Obese with metabolic syndrome (N = 20)	Obese without metabolic syndrome (N = 20)	Control (N = 12)
Sex, n (%)						
Male	10 (71.4)	6 (50.0)	6 (50.0)	14 (70.0)	12 (60.0)	4 (33.0)
Female	4 (28.6)	6 (50.0)	6 (50.0)	6 (30.0)	8 (40.0)	8 (66.7)
Race, n (%)						
White	10 (71.4)	9 (75.0)	9 (75.0)	18 (90.0)	16 (80.0)	10 (83.3)
Black or African American	3 (21.4)	3 (25.0)	3 (25.0)	2 (10.0)	4 (20.0)	2 (16.7)
Asian	1 (7.1)	1 (2.6)	1 (2.6)	0 (0.0)	0 (0.0)	0 (0.0)
Ethnicity, n (%)						
Hispanic or latino				1 (5.0)		
Non-hispanic/latino	14 (100.0)	12 (100.0)	12 (100.0)	19 (95.0)	20 (100.0)	12 (100.0)
Age at entry (years)						
Mean (SD)	60.6 (7.11)	64.0 (8.10)	57.1 (8.82)	59.1 (10.23)	52.0 (10.71)	60.9 (11.37)
Median (min, max)	62.0 (47, 72)	66.0 (48, 73)	56.0 (45, 70)	59.0 (41, 74)	51.5 (33, 72)	65.5 (34, 72)

Participants were separated into cohorts (poorly-controlled diabetes mellitus, well-controlled diabetes mellitus, morbid obesity, obese with MetS, obese without MetS) according to BMI and HbA1c constraints.

The mean BMI (± SD) for the morbidly obese cohort was 45.2 ± 9.12 kg/m^2^; obese with MetS was 33.8 ± 1.85 kg/m^2^; obese with no MetS was 34.4 ± 2.83 kg/m^2^; poorly-controlled diabetes mellitus was 30.4 ± 4.28 kg/m^2^; well-controlled diabetes mellitus was 26.5 ± 2.33 kg/m^2^, and the healthy patient group was 22.1 ± 1.27 kg/m^2^.

The mean HbA1c level was 10.1% in the poorly-controlled diabetes cohort, 6.3% in the well-controlled diabetes cohort, 5.6% in the morbidly obese, 5.9% in the obese with MetS, 5.5% in the obese without MetS, and 5.6% in the healthy patient cohort. Mean fasting blood glucose followed a similar pattern. In addition to those in the obese with MetS cohort, 85.7% in the poorly-controlled diabetes cohort and 58.3% each in the well-controlled diabetes and morbidly obese cohorts were also diagnosed with MetS.

### Immunogenicity results

No significant differences were noted between Visit 1 and Visit 2 for all parameters assessed; therefore data from both visits were pooled for all results.

#### PBMC and neutrophil cell counts

**Fig. 2:**
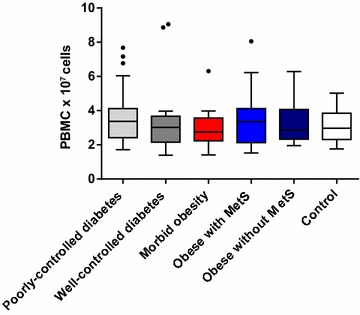
Mean PBMC cell count (× 10^7^ cells): both visits (Visit 1 and Visit 2) combined. *PBMC* peripheral blood mononuclear cell. The bottom and top edges of the box are located at the sample 25th and 75th percentiles and the center horizontal line is drawn at the 50th percentile (median). The whiskers extend at most 1.5 interquartile ranges

**Fig. 3:**
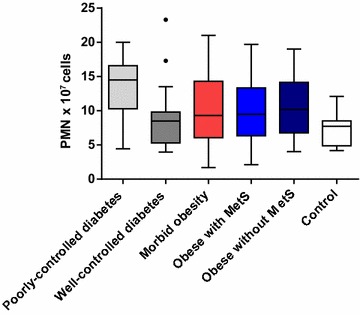
Mean neutrophil cell count (× 10^7^ cells): both visits (Visit 1 and Visit 2) combined. *PMN* polymorphonuclear neutrophil. The bottom and top edges of the box are located at the sample 25th and 75th percentiles and the center horizontal line is drawn at the 50th percentile (median). The whiskers extend at most 1.5 interquartile ranges

The average cell count of both visits ranged from 2.7 to 3.4 × 10^7^ for PBMCs and 7.1 to 12.1 × 10^7^ for neutrophils. The 95% CIs of the 6 cohorts overlapped for both PBMCs and neutrophil counts. Median PBMC and neutrophil cell counts were comparable for all 6 cohorts (Figs. 2, 3). These results indicate that there was no evidence that the number of PBMCs or neutrophils were overtly different between the cohorts, except for the poorly controlled diabetes group, which had a trend for a higher median number of neutrophils.

#### Chemotaxis

**Fig. 4:**
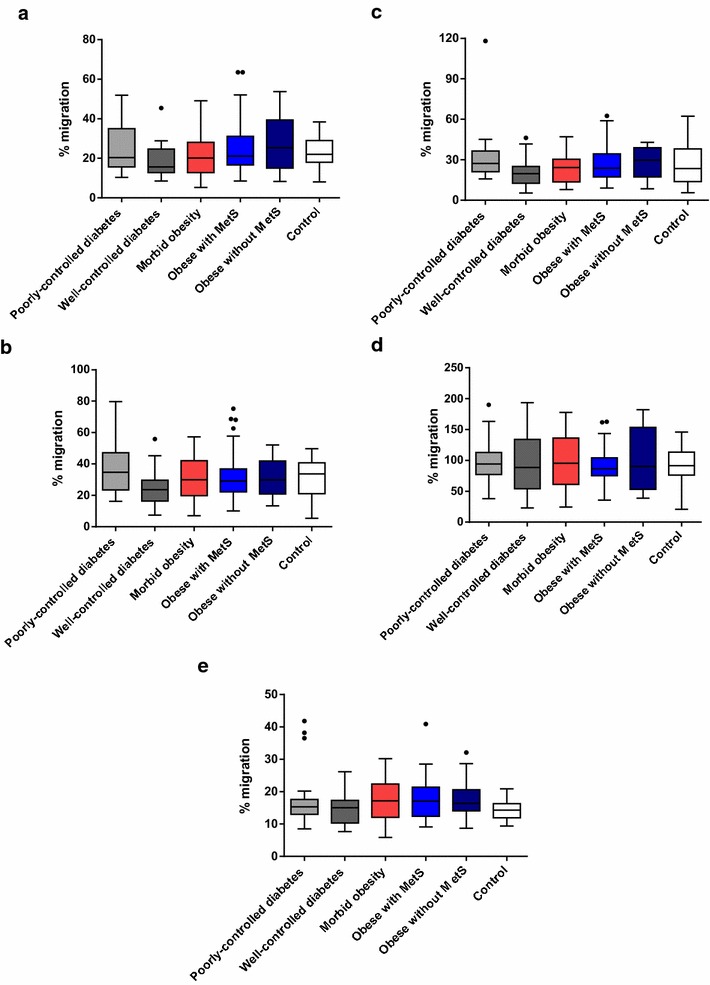
Mean % migration towards chemoattractants tested (fMLP, IL-8, C5a, FBS media) and RPMI media alone: both visits (Visit 1 and Visit 2) combined. **a** fMLP chemotaxis **b** IL-8 chemotaxis **c** C5a chemotaxis **d** FBS media chemotaxis **e** RPMI media chemotaxis. FBS: Fetal bovine serum. The bottom and top edges of the box are located at the sample 25th and 75th percentiles and the center horizontal line is drawn at the 50th percentile (median). The whiskers extend at most 1.5 interquartile ranges

The average percentage migration at both visits ranged from 17 to 25% for fMLP, from 25 to 36% for IL-8, from 20 to 29% for C5a, and from 83 to 103% for FBS (positive control chemoattractant). The 95% CIs of the 6 cohorts overlapped for fMLP, IL-8, C5a, and FBS, indicating no difference in % migration between the cohorts for any of the chemoattractants tested. The median migration for all 6 cohorts was comparable for the chemoattractants tested (Fig. 4). These results indicate that there was no evidence for differences in neutrophil migration rate towards chemoattractants fMLP, IL-8, and C5a for all 6 cohorts.

#### Phagocytosis

**Fig. 5:**
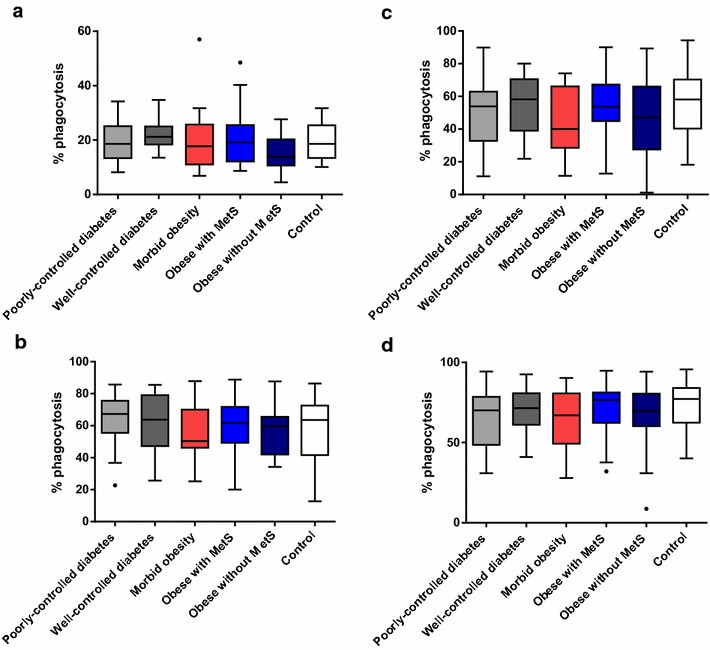
Mean % phagocytosis (neutrophil uptake of opsonized *S. aureus*) (no opsonization, individual plasma, normal human sera, CP8 immune sera): both visits (Visit 1 and Visit 2) combined. **a** No opsonization, **b** Individual plasma, **c** Normal human sera,** d** CP8 immune sera. The bottom and top edges of the box are located at the sample 25th and 75th percentiles and the center horizontal line is drawn at the 50th percentile (median). The whiskers extend at most 1.5 interquartile ranges.

The average % phagocytosis determined in both visits ranged from 50 to 64% for individual (autologous) plasma, and from 63 to 72% for the same exogenously provided CP8 immune sera. The 95% CIs of the 6 cohorts overlapped for individual autologous plasma samples, normal human sera, and CP8 immune sera, indicating no difference in % phagocytosis between the cohorts for any of the opsonizing agents tested. The 95% confidence interval of the % phagocytosis for all 6 cohorts overlapped for all opsonizing agents tested (Fig. 5). These results indicate no overt differences in neutrophil phagocytic capabilities for all of the opsonizing agents tested in these subgroups.

#### Neutrophil subsets by cohort

The average % neutrophil subsets range at both visits was 81–91% for normal neutrophils, 2–6% for suppressors, and 3–5% for killer neutrophils. The % CD47+ neutrophils ranged from 99.8 to 100%. The 95% CIs of the 6 cohorts overlapped for each of the neutrophil subsets and the median % of neutrophil subsets for all 6 cohorts were also not overtly different (Table 2). These results indicate that the neutrophils from the morbid and healthy patient cohorts had similar percentages of normal (CD16^bright^ CD62L^bright^), suppressor (CD16^bright^ CD62L^dim^), killer (CD16^dim^ CD62L^bright^), and CD47+ neutrophils.

**Table 2:** Neutrophil subsets (average %) by cohort of Visit 1 and Visit 2

Cohort						
Neutrophil subset	Poorly-controlled diabetes mellitus (N = 14)	Well-controlled diabetes mellitus (N = 12)	Morbid obesity (N = 12)	Obese with metabolic syndrome (N = 20)	Obese without metabolic syndrome (N = 20)	Control (N = 12)
“Normal” (CD16^bright^/CD62L^bright^)						
N, % RSD	14, 3.73	12, 7.54	12, 18.10	20, 9.11	20, 16.83	12, 6.85
Mean, median	91.2, 92.3	90.3, 91.9	81.1, 84.0	86.3, 88.4	84.0, 89.9	88.5, 89.0
95% CI	84.2, 98.9	76.5, 106.5	54.7, 120.5	71.3, 104.4	59.2, 119.2	76.1, 102.9
“Suppressors” (CD16^bright^/CD62L^dim^)						
N, % RSD	14, 107.5	12, 135.3	12, 150.9	20, 143.6	20, 213.2	12, 124.2
Mean, median	2.5, 2.5	2.4, 2.2	5.9, 6.1	3.4, 3.2	4.2, 3.6	2.2, 1.5
95% CI	0.4, 16.9	0.3, 22.7	0.5, 65.1	0.4, 31.1	0.3, 65.1	0.3, 18.5
“Killer” (CD16^dim^/CD62L^bright^)						
N, % RSD	14, 82.83	12, 83.71	12, 60.22	20, 78.80	20, 55.68	12, 43.27
Mean, median	2.8, 2.7	3.1, 3.1	4.9, 5.3	4.7, 5.1	3.0, 3.2	4.7, 4.9
95% CI	0.6, 13.3	0.6, 15.5	1.5, 16.8	1.1, 20.2	1.0, 8.9	1.9, 11.7
%CD47+						
N, % RSD	14, 0.03	12, 0.44	12, 0.04	20, 0.23	20, 0.04	12, 0.01
Mean, median	100.0, 100.0	99.8, 100.0	100.0, 100.0	99.9, 100.0	100.0, 100.0	100.0, 100.0
95% CI	99.9, 100.0	98.8, 100.7	99.9, 100.1	99.4, 100.4	99.9, 100.1	100.0, 100.0
CD47 MFI						
N, % RSD	14, 25.13	12, 48.26	12, 18.40	20, 17.28	20, 19.60	12, 19.80
Mean, median	18,462, 16,709	15,558, 16,598	19,060, 19,857	16,627, 17,062	17,226, 16,118	18,164, 17,718
95% CI	10,817, 31,510	5684, 42,590	12,757, 28,477	11,612, 23,809	11,475, 25,860	11,797, 27,967

*CI* confidence interval, *MFI* mean fluorescence intensity, *RSD* relative standard deviation

#### OPA titers

OPA geometric mean titers of both visits ranged from 13,014 to 27,569 for high titered CP8 immune sera, from 1001 to 1434 for medium titered CP8 immune sera, and from 59.7 to 87.3 for low titered CP8 immune sera. The 95% CIs of the OPA titers in the 6 cohorts overlapped irrespective of titer level (Table 3), so there was no evidence for differences in opsonophagocytosis activity among the neutrophils in all cohorts.

**Table 3:** Average OPA (titer) by cohort: both visits (Visit 1 and Visit 2) combined

Cohort						
Subset	Poorly-controlled diabetes mellitus (N = 14)	Well-controlled diabetes mellitus (N = 12)	Morbid obesity (N = 12)	Obese with metabolic syndrome (N = 20)	Obese without metabolic syndrome (N = 20)	Control (N = 12)
High titer sera						
N, % RSD	14, 84.23	12, 54.00	12, 187.7	18, 197.2	20, 427.0	12, 89.48
Mean, median	24,968, 28,580	27,569, 30,114	13,422, 20,032	17,408, 21,465	12,036, 23,405	13,014, 13,094
95% CI	5133, 121,462	9056, 83,928	898, 200,545	1220, 248,333	329, 440,071	2406, 70,391
Medium titer sera						
N, % RSD	14, 26.81	12, 85.08	12, 44.84	20, 81.37	20, 106.0	12, 25.00
Mean, median	1262, 1222	1434, 1366	1109, 1135	1204, 1089	1224, 1363	1001, 1013
95% CI	714, 2230	283, 7276	432, 2844	271, 5351	199, 7529	582, 1722
Low titer sera						
N, % RSD	14, 88.09	12, 40.61	12, 26.80	20, 93.19	20, 54.46	12, 65.10
Mean, median	87.3, 66.4	69.1, 63.4	59.7, 50.0	79.8, 55.4	77.5, 66.1	77.3, 63.8
95% CI	17.0, 448.9	29.2, 163.3	33.4, 106.5	15.2, 417.4	26.7, 225.1	20.9, 286.1

*CI* confidence interval, *OPA* opsonophagocytic assay, *RSD* relative standard deviation

#### cLIA titers

**Fig. 6:**
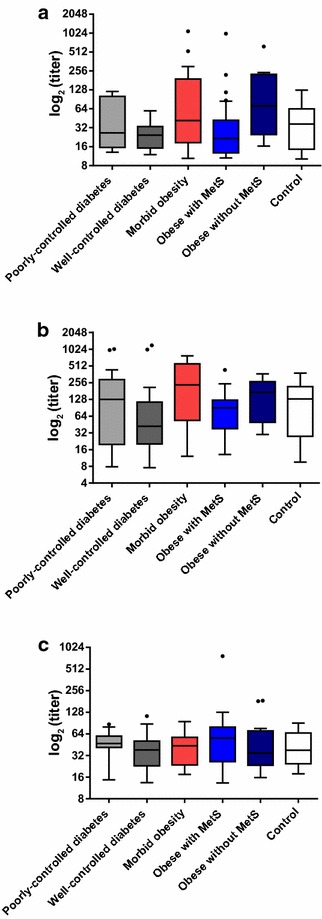
Mean CP5, CP8, and ClfA competitive Luminex immunoassay (cLIA) titers: both visits (Visit 1 and Visit 2) combined. **a** CP5 **b** CP8 **c** ClfA. The bottom and top edges of the box are located at the sample 25th and 75th percentiles and the center horizontal line is drawn at the 50th percentile (median). The whiskers extend at most 1.5 interquartile ranges

The average cLIA titer in subjects at both visits ranged from 24.9 to 55.5 for CP5, from 50.5 to 170 for CP8, and from 36.5 to 50.6 for ClfA. The 95% CIs of the 6 cohorts overlapped for the CP5, CP8, and ClfA cLIA. cLIA scatterplots per cohort for CP5, CP8, and ClfA show no evidence for substantial differences in the median cLIA titers for all cohorts (Fig. 6), indicating that the morbid cohorts had no overt differences in pre-existing CP5, CP8, and ClfA titers compared with the healthy patient cohort. An increase in the interquartile range of CP5 titers was noted in the morbid obesity and the obese without MetS cohorts, although the 95% confidence intervals were overlapping between these groups and the healthy patient cohort. There were too few subjects with an rMntC titer to allow for statistical analysis.

### Covariate results

Neutrophil assay results were summarized for the following variables with all cohorts pooled: fasting glucose (mg/dL); HbA1c (%); BMI (kg/m^2^); smoking; age; sex; CRP (mg/L); statin use; use of diabetes medication, and duration of diabetic history (days). Neutrophil function results were similar across all of these variables.

## *Post*-*hoc* analysis using alternative AHA/NHLBI diagnostic criteria for metabolic syndrome

### Subject disposition and characteristics

In this analysis, subjects were reassigned using the alternative AHA/NHLBI MetS criteria [28]; 8 subjects who were previously assigned to the ‘obese without MetS’ cohort were moved to the ‘obese with MetS’ cohort. This shift reflected the inclusion of individuals who were on medication to correct the MetS. Using the alternative criteria, there were 28 subjects assigned to the ‘obese with MetS’ cohort and 12 subjects assigned to the ‘obese without MetS’ cohort who completed Visit 1. Thirteen subjects in the ‘obese with MetS’ cohort and 10 subjects in the ‘obese without MetS’ cohort were brought in for Visit 2. The subject assignments to the remaining 4 cohorts were identical with those of the main analysis. The demographic characteristics for the ‘obese with MetS’ and the ‘obese without MetS’ cohorts are shown in Table 4.

**Table 4:** Subject demographics for analysis using AHA/NHLBI criteria metabolic syndrome criteria affecting the obese with metabolic syndrome cohort and obese without metabolic syndrome cohort

Cohort						
Characteristics	Poorly-controlled diabetes mellitus (N = 14)	Well-controlled diabetes mellitus (N = 12)	Morbid obesity (N = 12)	Obese with metabolic syndrome (N = 28)*	Obese without metabolic syndrome (N = 12)*	Control (N = 12)
Sex, n (%)						
Male	10 (71.4)	6 (50.0)	6 (50.0)	18 (64.3)	8 (66.7)	4 (33.0)
Female	4 (28.6)	6 (50.0)	6 (50.0)	10 (35.7)	4 (33.3)	8 (66.7)
Race, n (%)						
White	10 (71.4)	9 (75.0)	9 (75.0)	24 (85.7)	10 (83.3)	10 (83.3)
Black or African American	3 (21.4	3 (25.0)	3 (25.0)	4 (14.3)	2 (16.7)	2 (16.7)
Asian	1 (7.1)	0 (0.0)	0 (0.0)	0 (0.0)	0 (0.0)	0 (0.0)
Ethnicity, n (%)						
Hispanic or Latino	0 (0.0)	0 (0.0)	0 (0.0)	1 (3.6)	0 (0.0)	0 (0.0)
Non-Hispanic/Latino	14 (100.0)	12 (100.0)	12 (100.0)	27 (96.4)	12 (100.0)	12 (100.0)
Age at entry (years)						
Mean (SD)	60.6 (7.11)	64.0 (8.10)	57.1 (8.82)	58.7 (9.98)	48.1 (9.72)	60.9 (11.37)
Median (min, max)	62.0 (47, 72)	66.0 (48, 73)	56.0 (45, 70)	58.5 (41, 74)	48.5 (33, 70)	65.5 (34, 72)

*AHA/NHLBI* American Heart Association/National Heart, Lung, and Blood Institute

* Repeated analysis using AHA/NHLBI metabolic syndrome criteria [24] for obese with metabolic syndrome and obese without metabolic syndrome cohorts

The mean BMI (± SD) for the ‘obese with MetS’ was 34.1 ± 1.91 kg/m^2^ and ‘obese without MetS’ was 34.0 ± 3.33 kg/m^2^. This compares with a mean BMI of 33.8 ± 1.85 kg/m^2^ for the ‘obese with MetS’ and 34.4 ± 2.83 kg/m^2^ for the ‘obese without MetS cohort’ in the per protocol analysis. The mean HbA1c level was 5.8% in the ‘obese with MetS’ and 5.5% in the ‘obese without MetS’. In addition to those in the ‘obese with MetS’ cohort, 85.7% of the subjects in the poorly controlled diabetes, 83.3% of the morbidly obese and 75.0% of the well-controlled diabetes cohorts were also diagnosed with MetS.

### Immunogenicity results

Results for all immunogenicity parameters (PBMC/neutrophil counts, chemotaxis, phagocytosis, OPA, and cLIA titers) in the reassigned ‘obese without MetS’ and ‘obese with MetS’ cohorts were similar to the results of the per protocol analysis (data not shown).

## Discussion

This exploratory clinical research study showed no measurable impairment of ex vivo function in neutrophils isolated from 78 individuals with poorly or well-controlled diabetes, obesity, or MetS, compared with neutrophils from healthy patients.

It has been suggested that chronic low-grade inflammation, which is associated with increased blood counts of neutrophils, lymphocytes, and other inflammatory markers, may be involved in the pathogenesis of obesity, insulin resistance, and diabetes [29]. In this study, there was some variability in neutrophil counts between cohorts, with a trend towards a higher number of neutrophils in obese subjects, and in those with long-term diabetes, high CRP, high fasting glucose, and high HbA1c. These differences were not statistically significant, perhaps due to the relatively small numbers per cohort, as discussed later.

Neutrophils from patients with poorly-controlled, uninfected diabetes were reported to show reduced *S. aureus* killing compared with neutrophils from patients with well-controlled diabetes or those of healthy patients [30]. The altered neutrophil phagocytosis and bactericidal activity in poorly-controlled diabetes patients is thought to be associated with poor blood glucose control and may be related to the direct or indirect effects of insulin, as evidence shows that these activities can be restored by insulin administration [16, 30]. In our study, neutrophils from all cohorts showed similar ability to migrate towards chemotactic stimuli and phagocytose and kill *S. aureus*.

A recent small study of 30 obese patients with diabetes, hyperlipidemia, and high blood pressure found the functional capacity of neutrophils from these comorbid patients to be comparable with that of lean patients, in terms of phagocytosis, chemotaxis, and superoxide-generating capacity towards *Escherichia coli* [31]. No alterations in neutrophil functions were observed with differing age, gender, diabetic status, or hyperlipidemia [31]. In our study, similarly, neutrophil function was similar when grouped by sex, age, HbA1c, BMI, fasting glucose, smoking, use of statin or diabetes medication, by diabetes history, or when grouping patients using different MetS criteria (data not shown).

There were no significant differences in neutrophil subset populations or pre-existing antibody titers among all studied cohorts, as has been previously reported [12, 13]. Based on these results, a similar functional immune response to *S. aureus* antigens may be predicted in comorbid and healthy cohorts.

The main limitation of this study was that the number of patients sampled per cohort was small, which may reduce the ability to detect more subtle between-group differences in neutrophil counts and function. The lack of overt impairment in neutrophil activity seen in the comorbid cohorts in this in vitro study may differ from similar studies due to a number of factors, including study size, variability in experimental design and severity of disease, or level of diabetes control.

Reassignment of the ‘obese without MetS’ and the ‘obese with MetS’ cohorts based on the AHA/NHLBI criteria for MetS [28] showed no differences in neutrophil functional properties between the subjects with obesity and metabolic syndrome/obesity without MetS and the other cohorts, indicating that control of MetS with appropriate therapeutics does not overtly impact neutrophil function.

## Conclusions

The lack of measurable impairment in in vitro neutrophil function in individuals with obesity, MetS, or diabetes predicts that an efficacious *S. aureus* vaccine able to generate robust antibody responses that can enhance neutrophil-mediated bacterial killing in healthy subjects could, in principle, be efficacious in these comorbid populations. The safety, tolerability, and immunogenicity of the SA4Ag vaccine is currently under evaluation in patients undergoing elective spinal fusion surgery (NCT02388165).

## Abbreviations

AHA/NHLBI: American Heart Association/National Heart, Lung, and Blood Institute
BMI: body mass index
cLIA: competitive Luminex^®^ immunoassay
CRP: C-reactive protein
FBS: fetal bovine serum
MetS: metabolic syndrome
MFI: mean fluorescence intensity
OPA: opsonophagocytic activity
PBMC: peripheral blood mononuclear cell
RSD: relative standard deviation
*S. aureus*: *Staphylococcus aureus*
*SA3Ag*: *3*-*antigen S. aureus*
*SA4Ag*: *4*-*antigen S. aureus*
