# Correction: Neutrophil killing of *Staphylococcus aureus* in diabetes, obesity and metabolic syndrome: a prospective cellular surveillance study

**DOI:** 10.1186/s13098-024-01314-y

**Published:** 2024-03-25

**Authors:** Ingrid Lea Scully, Lisa Kristin McNeil, Sudam Pathirana, Christine Lee Singer, Yongdong Liu, Stanley Mullen, Douglas Girgenti, Alejandra Gurtman, Michael W. Pride, Kathrin Ute Jansen, Paul L. Huang, Annaliesa S. Anderson

**Affiliations:** 1 Pfizer Vaccine Research and Development, 401 North Middletown Rd, 10965 Pearl River, NY USA; 2 Cardiovascular Research Center and Cardiology Division, Department of Medicine, Massachusetts General Hospital and Harvard Medical School, Boston, MA USA

**Correction: Diabetol Metab Syndr (2017) 9:76**

10.1186/s13098-017-0276-3

After publication we became aware that the Acknowledgements section in our article [1] was incomplete. It should read as follows:

“Medical writing support was provided by Sharmila Blows, Ph.D., of Engage Scientific Solutions, and was funded by Pfizer. Johanna Hoyos, a former scientist at Pfizer-Wyeth, developed and performed the chemotaxis assay.”
